# Relationship between corneal cutting ratio and early postoperative *Q* values and higher-order aberrations after small incision lenticule extraction in myopic patients

**DOI:** 10.1097/MD.0000000000051344

**Published:** 2026-11-06

**Authors:** Zhuoqiong Wang, Wenpeng Dong, Qun Zhang, Guinan Chen

**Affiliations:** a Department of Ophthalmology, The Affiliated People’s Hospital of Ningbo University, Ningbo, Zhejiang, China.

**Keywords:** cutting ratio, femtosecond laser surgery, HOAs, *Q* value

## Abstract

This study investigates the association between the corneal cutting ratio and early postoperative corneal *Q* values and higher-order aberrations (HOAs) after small incision lenticule extraction (SMILE) in myopic patients. A total of 149 patients (288 eyes) with refractive errors who underwent SMILE in our hospital from January 2017 to December 2021 were included. Both eyes were included when they met the eligibility criteria; therefore, the eye was used as the unit of analysis. According to the cutting ratio (corneal cutting depth/preoperative corneal thickness), eyes were divided into the ≤0.16 group (n = 96) and >0.16 group (n = 192). Preoperative and 1-month postoperative uncorrected visual acuity (UCVA), best corrected visual acuity (BCVA), corneal HOAs, and corneal *Q* values were compared. Pearson correlation analysis was used to assess the associations between cutting ratio and corneal optical parameters. There were no significant differences in baseline refractive parameters or preoperative BCVA, total HOAs, and corneal *Q* values between the 2 groups (all *P* > .05), and no significant differences in postoperative UCVA or BCVA (*P* > .05). The >0.16 group had significantly higher total HOAs and corneal *Q* values at 1 month than the ≤0.16 group (*P* < .05). The cutting ratio was positively correlated with total HOAs and corneal *Q* values (*R* = 0.462 and 0.432, respectively; both *P* < .001). At 1 month after SMILE, a higher cutting ratio was associated with higher corneal *Q* values and HOAs. Because bilateral eyes from some patients were included and follow-up was limited to 1 month, these findings should be interpreted as early postoperative associations rather than evidence of a long-term visual-quality outcome.

## 1. Introduction

Small incision lenticule extraction (SMILE) is a femtosecond laser refractive procedure used to correct myopia and myopic astigmatism.^[1,2]^ During SMILE, a stromal lenticule is created with a femtosecond laser and removed through a small corneal incision, thereby changing corneal refractive power.^[3,4]^ This surgery is suitable for patients with myopia of 1000 to 1200 degrees or less and astigmatism of 400 to 600 degrees or less.^[5]^ At the same time, the patient’s corneal shape and thickness must meet the surgical requirements. The *Q* value is an important indicator for measuring corneal spherical aberration and is directly related to the clarity and comfort of postoperative vision.^[6]^ Higher-order aberrations (HOAs) cover all aberrations other than spherical and cylindrical aberrations and also have an important impact on visual quality. In femtosecond laser surgery, different cutting ratios may lead to changes in corneal shape and refractive state, thereby affecting the *Q* value and HOAs. The present study, therefore, investigated the association between cutting ratio and corneal *Q* values and HOAs at 1 month after SMILE. The study was designed to describe early postoperative optical changes and not long-term visual-quality outcomes.

## 2. Materials and methods

### 2.1. General information

This study was approved by the Ethics Committee of Jining Hospital of The Affiliated People’s Hospital of Ningbo University. A total of 149 patients (288 eyes) with refractive errors who underwent SMILE in our hospital from January 2017 to December 2021 were selected. Both eyes of a patient were included when both eyes satisfied the inclusion and exclusion criteria; otherwise, only the eligible eye was included. Thus, the eye was the unit of analysis. Inclusion criteria were: age 18 to 50 years; refractive power relatively stable during the previous 2 years, with an annual change of <0.50 D; myopia or myopic astigmatism; myopia within 10.00 D; corneal thickness >480 μm; complete clinical data; and informed agreement to participate. Exclusion criteria were: inability to complete routine surgery or preoperative examinations; unstable refraction; corneal disease, severe dry eye, ocular trauma, or previous corneal transplantation; insufficient corneal thickness; active ocular lesions or infection; severe eyelid defects, deformity, or eyelid closure insufficiency; and inability to cooperate with surgery. According to the cutting ratio (corneal cutting depth/preoperative corneal thickness), eyes were divided into the ≤0.16 group (n = 96) and >0.16 group (n = 192). The general data of the 2 groups were compared (*P* > .05), as shown in Table 1.

**Table 1:** Comparison of general data between the 2 groups [(±*s*) (n, %)].

Indicators	≤0.16 group (n = 96)	0.16 group (n = 192)	*t/x* ^*2*^	*P*
Age (yr)	27.75 ± 6.25	26.12 ± 6.44	0.469	.640
BMI (kg/m^2^)	22.28 ± 2.42	21.42 ± 2.25	0.474	.636
Sphere power (D)	−4.24 ± 1.03	−6.32 ± 1.05	0.589	.016
Cylinder power (D)	−0.81 ± 0.59	−1.12 ± 0.57	0.274	.784
Optical zone diameter (mm)	6.43 ± 0.72	6.32 ± 0.68	0.566	.572
Equivalent spherical refractive power (D)	−4.62 ± 1.82	−6.72 ± 1.79	0.467	.410

BMI = body mass index, D = dioptre.

### 2.2. Methods

#### 2.2.1. Preoperative preparation

All patients underwent the following routine preoperative examinations: using the Japanese Topcon automatic refractometer (TOPCON), slit lamp microscopy examination, non-contact tonometry measurement of non-contact intraocular pressure, and Pentacam anterior segment analysis system to measure corneal topography thickness, corneal *Q* value, and HOAs.

#### 2.2.2. Surgical procedure

Before the surgery, the patients’ eyes were routinely disinfected. 3 times of surface anesthesia with 0.5% procaine hydrochloride eye drops (s.a. ALCON-COUVREURn.v., National Drug Approval Number HJ20160133, 15 mL: 75 mg/branch), 5 minutes each time. The patients lay flat, a sterile drape was laid, an eyelid opener was used to help the patients keep their eyelids open, the negative pressure suction ring was connected to the laser emission window and the treatment control panel, and it was placed on the patient’s temporal side. The patient’s head position was adjusted, and the patient was instructed to gaze at the light. After confirming that the surgical preparations were complete, the corneal scanning cutting frequency of the VisualMax femtosecond laser was set to 500 kHz, the cutting energy was controlled at 150 nJ, the corneal cap thickness was 120 μm, the corneal cap diameter was 7.6 mm, and the optical zone was 6.5 mm. For patients in the ≤0.16 group, the cutting ratio was ≤0.16, and for patients in the >0.16 group, the cutting ratio was >0.16. The ultra-short laser pulses precisely emitted by the laser system finely carved the corneal stroma layer according to the preset refractive correction algorithm, shaping a lens-like tissue layer that met the requirements. With the help of precise instruments, the custom corneal stromal lens was safely removed. Through sufficient rinsing with balanced salt solution, the corneal stromal pocket was deeply cleaned and flattened to ensure the optimization and recovery of the corneal shape.

#### 2.2.3. Postoperative medication and follow-up

On the first day after the surgery, most patients used Tobramycin Dexamethasone Eye Drops (Chengdu Hengrui Pharmaceutical Co., Ltd., National Drug Approval Number H20093842, specification: 5 mL: 15 mg: 5 mg), 1 drop per time, 4 times per day; Levofloxacin Eye Drops (Suzhou Industrial Park Tianlong Pharmaceutical Co, Ltd, National Drug Approval Number H20173405, specification: 5 mL: 24.4 mg), 1 drop per time, 4 times per day; and Hyaluronic Acid Eye Drops (Beijing Huanheng Land Pharmaceutical Co, Ltd, National Drug Approval Number H20193204, specification: 0.8 mL: 2.4 mg), 1 drop per time, 4 times per day. Both groups used the medication for 1 month and underwent uncorrected visual acuity (UCVA), best corrected visual acuity (BCVA), intraocular pressure, and Pentacam anterior segment analysis system examinations, etc.

### 2.3. Statistical methods

Data were analyzed using SPSS version 26.0. Continuous variables were expressed as mean ± standard deviation and compared between groups using independent-samples *t* tests; categorical variables were expressed as percentages and compared using chi-square tests. Pearson correlation coefficients were used to analyze the associations between cutting ratio and total HOAs and corneal *Q* values. A 2-sided *P* < .05 was considered statistically significant, and *P* values smaller than .001 are reported as *P* < .001 rather than *P* = .000. Because both eyes of some participants were included and the available analysis treated eyes as individual observations, within-subject inter-eye correlation was not explicitly modeled; this issue is acknowledged as a methodological limitation.

## 3. Results

### 3.1. Comparison of indicators before and after surgery in both groups

There were no significant differences in preoperative BCVA, total HOAs, and corneal *Q* values between the 2 groups (*P* > .05); postoperative comparisons of UCVA and BCVA between the 2 groups also showed no significant differences (*P* > .05). The total HOAs and corneal *Q* values in the >0.16 group were significantly higher than those in the ≤0.16 group, and the differences between the groups were significant (*P* < .05; see Table 1).

### 3.2. Correlation analysis of cutting ratio with total HOAs and corneal Q value

Pearson correlation analysis showed that the cutting ratio was positively correlated with total HOAs and corneal *Q* value at 1 month (*r* = 0.462 and 0.432, respectively; both *P* < .001). These correlations indicate an association and should not be interpreted as demonstrating an independent causal effect of cutting ratio, particularly given the baseline refractive imbalance and the non-independence of bilateral-eye data (see Table 2).

**Table 2:** Comparison of various indicators before and after surgery in the 2 groups (±*s*).

Group	Number of examples	UCVA		BCVA		总HOAs		Corneal *Q* value	
		Before the operation	After the operation	Before the operation	After the operation	Before the operation	After the operation	Before the operation	After the operation
≤0.16 group	96	0.31 ± 0.03	0.95 ± 0.13	1.16 ± 0.12	1.17 ± 0.15	0.280 ± 0.072	0.386 ± 0.132	−0.18 ± 0.16	0.19 ± 0.18
Group 0.16	192	0.12 ± 0.09	0.93 ± 0.11	1.14 ± 0.14	1.15 ± 0.14	0.256 ± 0.081	0.483 ± 0.125	−0.17 ± 0.17	0.40 ± 0.31
*t*	–	1.393	1.294	1.260	1.090	0.124	-4.890	0.490	7.254
*P*	–	.032	.198	.209	.277	.901	.000	.625	.000

BCVA = best corrected visual acuity, HOAs = higher-order aberrations, UCVA = uncorrected visual acuity.

## 4. Discussion

With the rapid development of technology, femtosecond laser surgery has become one of the important methods for correcting myopia.^[7]^ This technology, with its advantages such as small trauma, quick recovery, and high safety, has been widely applied in the field of ophthalmology. However, the cutting ratio in femtosecond laser surgery, that is, the ratio of corneal cutting depth to preoperative corneal thickness, has always been a hot topic in ophthalmological research.^[8]^ In particular, the relationship between the cutting ratio and the *Q* value and HOAs of myopic patients after surgery has attracted much attention. Studies have shown that the size of the cutting ratio is significantly correlated with the increase in postoperative *Q* value and HOAs.^[9,10]^ The larger the cutting ratio, the more obvious the increase in postoperative corneal *Q* value and HOAs, which affects the visual quality of patients.^[11,12]^ This study selected 149 patients with refractive errors who underwent femtosecond laser surgery in our hospital from January 2017 to December 2021, totaling 288 eyes. The aim was to explore the relationship between different cutting ratios of femtosecond laser surgery and the postoperative *Q* value and HOAs of myopic patients, in order to provide new ideas and methods for the development of femtosecond laser surgery and bring more safe and effective surgical treatment options to myopic patients.

In this study, there was no significant difference in preoperative UCVA, BCVA, total HOAs, and corneal *Q* value between the 2 groups (*P* > .05). There was also no significant difference in postoperative UCVA and BCVA between the 2 groups (*P* > .05). However, the total HOAs and corneal *Q* value in the >0.16 group were significantly higher than those in the ≤0.16 group, and the differences between the groups were significant (*P* < .05). Further Pearson correlation analysis showed that the cutting ratio was positively correlated with total HOAs and corneal *Q* value (*r* = 0.462, 0.432; *P* = .000, .000). This suggests that the cutting ratio of femtosecond laser surgery is closely related to *Q* value, HOAs, and spherical aberration. The larger the cutting ratio, the more obvious the increase in postoperative *Q* value and HOAs, and the greater the impact on the visual quality of patients. Corneal *Q* value is an important indicator for measuring the non-spherical characteristics of the cornea, reflecting the difference in curvature radii at the center and edge of the cornea, which is crucial for visual quality in low-light and dim-light conditions.^[13]^ Corneal *Q* value can make the corneal shape more in line with the standard state of the human eye, reduce or avoid spherical aberration that may be introduced by refractive surgery, and thereby improve postoperative visual quality. In femtosecond surgery, the selection of the cutting ratio directly determines the amount and position of corneal cutting, and thereby affects the *Q* value of the postoperative cornea.^[14]^ Studies have shown^[15]^ that an excessively high cutting ratio may lead to excessive corneal cutting, causing changes in the posterior surface morphology of the cornea and thereby affecting the *Q* value, resulting in an increase in postoperative spherical aberration and a decline in visual quality. On the contrary, an excessively low cutting ratio may not be able to fully correct myopia and affect the surgical outcome. Therefore, there is a positive correlation between the cutting ratio of femtosecond surgery and the postoperative corneal *Q* value. HOAs refers to the total sum of all aberrations other than spherical and cylindrical aberrations, and it plays a crucial role in visual quality. In femtosecond surgery, the cutting ratio determines the depth and range of corneal cutting, and thereby affects the optical performance of the postoperative cornea, including changes in HOAs.^[16]^ If the cutting ratio is selected appropriately, it can not only fully correct myopia but also preserve the natural shape of the cornea to the greatest extent, resulting in a relatively smaller increase in HOAs, which not only improves the patient’s naked eye vision but also improves their visual comfort and reduces the occurrence of postoperative complications.^[17]^ When the cutting ratio is too large, it means that more of the cornea is being cut, which may cause the corneal surface to become more uneven, introducing more HOAs. This will interfere with the focusing process of light within the eye, leading to a decline in postoperative visual quality, such as the appearance of phenomena like glare and halos.^[18]^ Therefore, there is a positive correlation between the cutting ratio of LASIK surgery and postoperative HOAs. Clinical practice requires precise calculation of the cutting ratio to ensure that the *Q* value and HOAs of the cornea are within controllable ranges, thereby balancing the relationship between the correction effect and visual quality.^[19,20]^

The present findings require cautious interpretation. Both eyes from some participants were included, whereas the available eye-level statistical analysis did not explicitly account for within-subject inter-eye correlation. This may affect the precision of standard errors and *P* values. Future studies should therefore consider patient-clustered methods, generalized estimating equations, or mixed-effects models to account for inter-eye dependence.

In addition, the postoperative assessment in this study was limited to 1 month. Corneal healing, epithelial and stromal remodeling, and optical quality can continue to change beyond the early postoperative period. Accordingly, the present results describe early postoperative corneal optical characteristics and cannot be extrapolated to long-term visual quality or refractive stability. Longer prospective follow-up with repeated measurements is needed to determine whether the observed associations persist over time. In conclusion, at 1 month after SMILE, a higher cutting ratio was associated with higher corneal *Q* values and total HOAs. Given the inclusion of bilateral eyes in some participants, the single-center design, and the short follow-up, the findings should be interpreted as early postoperative associations and require confirmation in longer-term studies.

In conclusion, the cutting ratio in SMILE was positively associated with the early postoperative changes in corneal *Q* value and total higher-order aberrations in myopic patients. A higher cutting ratio was associated with greater increases in corneal *Q* value and HOAs at 1 month after surgery. These findings suggest that the cutting ratio may be an important factor to consider when planning SMILE procedures, while maintaining adequate corneal safety and refractive correction. However, the present study was conducted at a single center with a relatively limited sample size and a short follow-up period of only 1 month. In addition, the inclusion of both eyes from some patients may introduce inter-eye correlation. Therefore, larger multicenter studies with longer follow-up and statistical methods accounting for within-subject correlation are needed to confirm the long-term relationship between cutting ratio, corneal optical quality, and postoperative visual outcomes.

## Author contributions

**Conceptualization:** Zhuoqiong Wang, Wenpeng Dong, Qun Zhang, Guinan Chen.

**Data curation:** Zhuoqiong Wang, Wenpeng Dong, Qun Zhang, Guinan Chen.

**Formal analysis:** Zhuoqiong Wang, Wenpeng Dong, Qun Zhang, Guinan Chen.

**Funding acquisition:** Zhuoqiong Wang.

**Writing – original draft:** Zhuoqiong Wang.

**Writing – review & editing:** Zhuoqiong Wang.
